# Immortalized B Cells Transfected With mRNA of Antigen Fused to MITD (IBMAM): An Effective Immunological Tool for In-Vitro Immune-Monitoring of Antigen-specific T Cells

**DOI:** 10.1200/GO.22.27000

**Published:** 2022-05-05

**Authors:** Tiantian Zhang, Zhe Wang, Soung-Chul Cha, Jingwei Liu, Zhenyuan Dong, Szymon Szymura, Aaron Anderson, Benjamin Kuang, Sattva Neelapu, Larry Kwak

**Affiliations:** ^1^ City of Hope National Medical Center, Duarte, CA; ^2^ University of Texas MD Anderson Cancer Center, Houston, TX

## Abstract

To overcome technical limitations of detecting low frequencies of antigen-specific-T cells in human patients, we sought to establish a highly sensitive platform for antigen-specific-T cell detection and activation using IBMAM (immortalized B cells transfected with mRNA of antigen fused to MHC-class-I-trafficking-domain [MITD]).

**METHODS:**

We first established immortalized B cells in-vitro as antigen-presenting cells. B cells were isolated from CMV-seropositive normal healthy donors (NHDs), stimulated, and transduced with retrovirus containing BCL-6 and BCL-XL. Purified CD4+ T lymphocytes obtained from the NHDs PBMC were co-cultured in-vitro with autologous immortalized B cells transfected with CMVpp65 mRNA fused to MITD (IBMAM) as a model antigen and then measured for activation using IFN-g ELISA. We developed two pathways for IBMAM mediated antigen-specific T cell activation and detection. For the first pathway, if IFN-g is detected, T cells are isolated again from NHDs PBMC and expanded using rapid expansion procedures (REP). After the first REP, antigen-specific T cells are detected using IBMAM, sorted, and a second REP is performed. However, for the second pathway, if IFN-g is not detected, NHDs PBMC is stimulated twice using IBMAM and subsequently antigen-specific T cells are detected using IBMAM again.

**RESULTS:**

B cells were successfully immortalized by transfecting at least 5*105 cells with retrovirus (1MOI) and co-cultured with CD40L-IL21 expressing human fibroblasts. We observed a significantly increased activation of antigen-specific CD4+ T cells when using IBMAM in comparison to controls transfected with CMVpp65 mRNA lacking the MITD fusion or a known CMVpp65 peptide pool. For pathway 1 and 2 of our platform, successful detection, activation, and expansion of CMVpp65-specific T cells were observed from low frequency NHDs samples using IBMAM.

**CONCLUSION:**

We have thus established a highly sensitive method of immuno-monitoring that can be used for the detection of antigen-specific T cells in patient samples with low frequencies of antigen-specific-T cells, such as human cancer antigens.

